# A Perspective on the Role of Metformin in Treating Myalgic Encephalomyelitis/Chronic Fatigue Syndrome (ME/CFS) and Long COVID

**DOI:** 10.1021/acsptsci.5c00229

**Published:** 2025-09-11

**Authors:** David Fineberg, Alain Moreau, Elena K. Schneider-Futschik, Christopher W. Armstrong

**Affiliations:** † Department of Biochemistry and Pharmacology, School of Biomedical Sciences, Faculty of Medicine, Dentistry and Health Sciences, 2281 The University of Melbourne, Parkville, Victoria 3010, Australia; ‡ Departement of Stomatology, Faculty of Dentistry, Université de Montréal, Montreal, Québec H3T 1J4, Canada; § Departement of Biochemistry and Molecular Medicine, Faculty of Medicine, Université de Montréal, Montreal, Québec H3T 1J4, Canada; ∥ Open Medicine Foundation ME/CFS Collaborative Research Center at the CHU Sainte-Justine/Université de Montréal, Montreal, Québec H3T 1C5, Canada

**Keywords:** ME/CFS, long COVID, myalgic encephalomyelitis, mitochondria, microbiome, metformin

## Abstract

Myalgic encephalomyelitis/chronic fatigue syndrome (ME/CFS) and Long COVID (LC) are increasingly recognized as debilitating postinfectious conditions that impact both individuals and society. Recent research highlights the potential of metformin, an antidiabetic agent, as a treatment for these syndromes by targeting their underlying mechanisms. This review assesses the effectiveness of metformin in ME/CFS and LC, which involve complex dysfunctions related to cytokines, glycolysis, ATP generation, oxidative stress, gastrointestinal microbiomes, and vascular endothelial function. Metformin, traditionally known for its antihyperglycemic properties may offer broader therapeutic benefits by influencing these pathological pathways. It works by inhibiting complexes I and IV of the electron transport chain, which reduces the strain on malfunctioning complex V and decreases the production of harmful free radicals. Additionally, metformin’s impact on mTOR signaling could improve energy metabolism in ME/CFS and LC by downregulating an overactive but underperforming protein, thereby alleviating symptoms. Beyond the impact on cellular metabolism, metformin has shown to have anti-inflammatory, vascular, gastrointestinal, neuroprotective and epigenetic effects. We explore this impact of metformin and the potential role it could play to help people with ME/CFS. While metformin shows promise, it is unlikely to be a stand-alone solution. Instead, it may be part of a broader treatment strategy that includes other therapies targeting neurocognitive and autonomic impairments.

## Graphical abstract

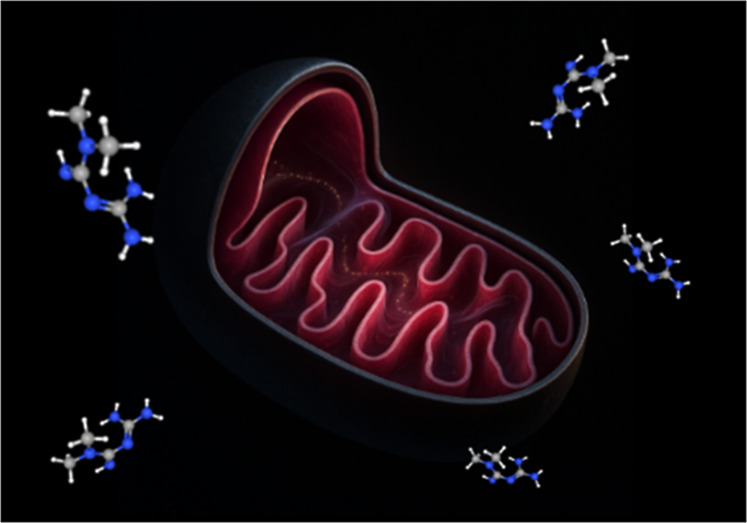

Myalgic encephalomyelitis/chronic fatigue syndrome (ME/CFS) is a complex debilitating illness primarily characterized by extreme fatigue, cognitive impairment (brain fog), post exertional malaise (PEM) and sleep disturbances.^1–6^ Despite estimates of 1.7–3.38 million people being affected in the United States alone,^7–9^ there are no specific diagnostic tests or treatments to date. ME/CFS is a serious life-changing condition,^10^ with a high degree of psychosocial impact. 54% report being unemployed and 87% note a serious impact on social life,^11^ with similar rates seen in Long COVID (LC).^12^ Up to 25% of those who suffer from ME/CFS are bed-bound.^13^ LC, a clinical syndrome and diagnosis of exclusion, is defined by persistent functionally impairing symptoms lasting longer than three months, following a confirmed acute COVID-19 infection.^14^ Key symptoms shared between ME/CFS and LC, such as fatigue, PEM, cognitive impairments, and sleep disturbances, contribute to the clinical heterogeneity of both conditions. The presence of comorbid conditions, including Mast Cell Activation Syndrome (MCAS), Postural Orthostatic Tachycardia Syndrome (POTS), Fibromyalgia (FM), and mood disorders, further complicates their treatment. Treatment of ME/CFS so far have focused on overall symptom improvement. In this review, we discuss a new perspective on therapy targeting and explore the mechanisms behind the potential use of metformin as a treatment for ME/CFS and LC, via its anti-inflammatory properties and impact on cellular metabolism, microbiome and epigenetic factors.

## Origins of Metformin

Metformin is a widely used first-line oral medication for type 2 diabetes, which controls blood glucose levels by decreasing glucose production in the liver and improving insulin sensitivity in peripheral tissues.^15,16^ It reduces cardiovascular disease and improves lipid profiles,^15,17,18^ as well as increasing performance in Cardiopulmonary Exercise Testing,^19^ an effect potentially transferrable to ME/CFS.^20^ Metformin has also been suggested as an anti-infective due to its multiple mechanisms of action.^21,22^ Previous studies indicate that metformin can inhibit the replication of certain viruses, reduce bacterial virulence, and enhance the host’s immune response.^23–26^ By modulating the gut microbiota and reducing systemic inflammation, metformin may also help in managing infections indirectly. These multifaceted benefits position metformin as a promising adjunctive therapy in the treatment of various infectious diseases. Indeed, originally used to treat influenza under the name Flamamine,^26^ metformin has been repurposed in acute COVID-19 treatment and as a vaccine adjuvant.^25,27^

While metformin has numerous benefits, there are several complications of varying severity. In 2016, the FDA issued a ‘black box warning’ for the development of life-threatening lactic acidosis.^28^ Minor complications are also prevalent in dose-dependent side effects such as diarrhea, nausea and abdominal pain in 20% of cases.^29^ Nabrdalik et al.’s systematic review found an almost 50% increased chance of abdominal pain compared with controls with a relative risk (RR) of 1.491 [1.211–1.836] (*p* = 0.0001), nausea was increased (RR 1.641, [1.169, 2.302], *p* = 0.0004), diarrhea, however, was seen as the most likely side effects (RR 2.445 [1.656–3.609] *p* = 0.0001).^29^

## Mechanisms of Action

Metformin acts through multiple mechanisms, targeting cellular receptors and intracellular pathways to regulate various metabolic processes.^30^ It is absorbed from the small intestine and moves intra- and extracellularly by organic cation transporters (OCT).^31,32^ Metformin is primarily eliminated through the kidneys, with a half-life of approximately 5 h.^33^ In hepatic cells, Adenosine monophosphate-activated protein kinase (AMPK) is activated by metformin, resulting in reduced glucose output and increased GLUT-4 translocation in adipocytes^34,35^ (see Figures 1 and 2). In addition to GLUT-1 and GLUT-4, metformin has also been shown to translocate GLUT-2 from apical to basolateral walls of the gut, reducing GI glucose uptake.^36^

**Figure 1:**
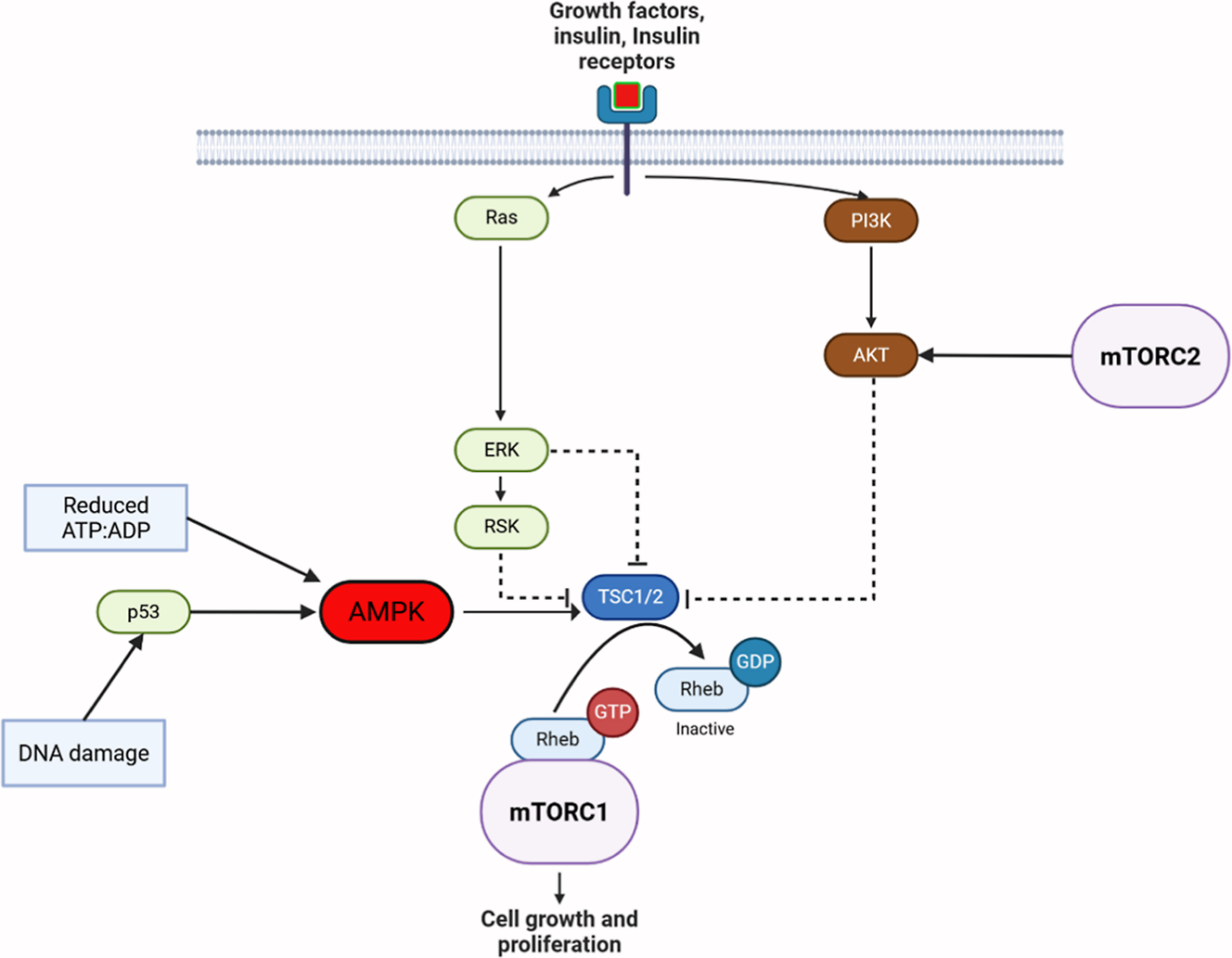
mTOR interacting with IR, AMPK and AMP. Triggering growth factors and insulin stimulate receptors on the cell membrane, activating Ras and PI3K. These pathways reduce TSC1/2 dephosphorylation and inactivation of mTORC1. The Ras pathway results in inhibitory activity of ERK and RSK on TSC1/2, while the PI3K/AKT pathway can be independently activated by mTORC2. TSC1/2 is responsible for the deactivation of mTORC1 by dephosphorylating and dissociating Rheb, which remains reversibly available. DNA damage and reduced intracellular energy stores accelerate the inhibitory activity of TSC through p53 and direct activity on AMPK. mTORC1 modulates expression of proteins responsible for cell growth and proliferation.

**Figure 2:**
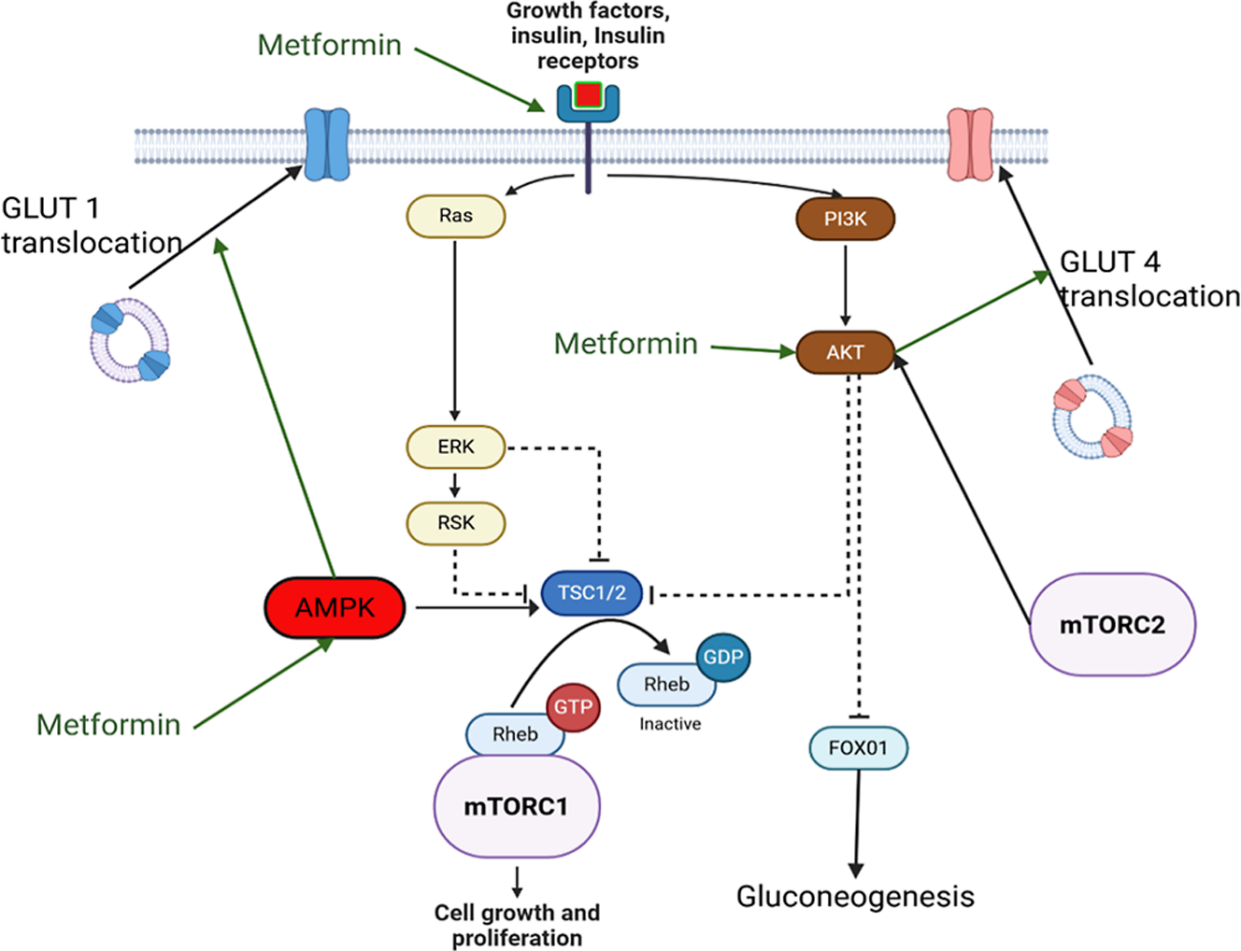
Metformin activities on the mTOR pathway. Metformin activating AMPK assists in translocating GLUT-1 to the cell surface and increases TSC1/2 activation. Metformin also activates AKT, resulting in GLUT-4 translocation to cell membrane and reduction in gluconeogenesis mediated by FOX01.

Metformin can exert its antihyperglycemic effects through mechanisms beyond glucose transporter proteins. Natali et al.’s meta-analysis showed strong inhibition of gluconeogenesis in addition to metformin’s actions on GLUT proteins.^37^ It has also been demonstrated that metformin’s antihyperglycemic actions involve inhibition of mitochondrial protein complex 1, which reduces hepatic glutamate oxidation while preserving succinate^38^ (see Figures 3 and 4). Metformin inhibits mitochondrial complex 1 and glycerophosphate dehydrogenase (mGPDH),^16^ while promoting AMP accumulation and activating AMPK.^39–41^ This leads to decreased hepatic glucose production, as well as reduced levels of fatty acids and triglycerides while increasing RNA encoding proteins involved in central insulin mediation.^42^ Another well-established antihyperglycemic action of metformin involves its accumulation of AMP, which inhibits adenylate cyclases.^43^ The decrease in cyclic AMP levels results in a reduction of glucagon-dependent glucose output from hepatocytes.^43^

**Figure 3:**
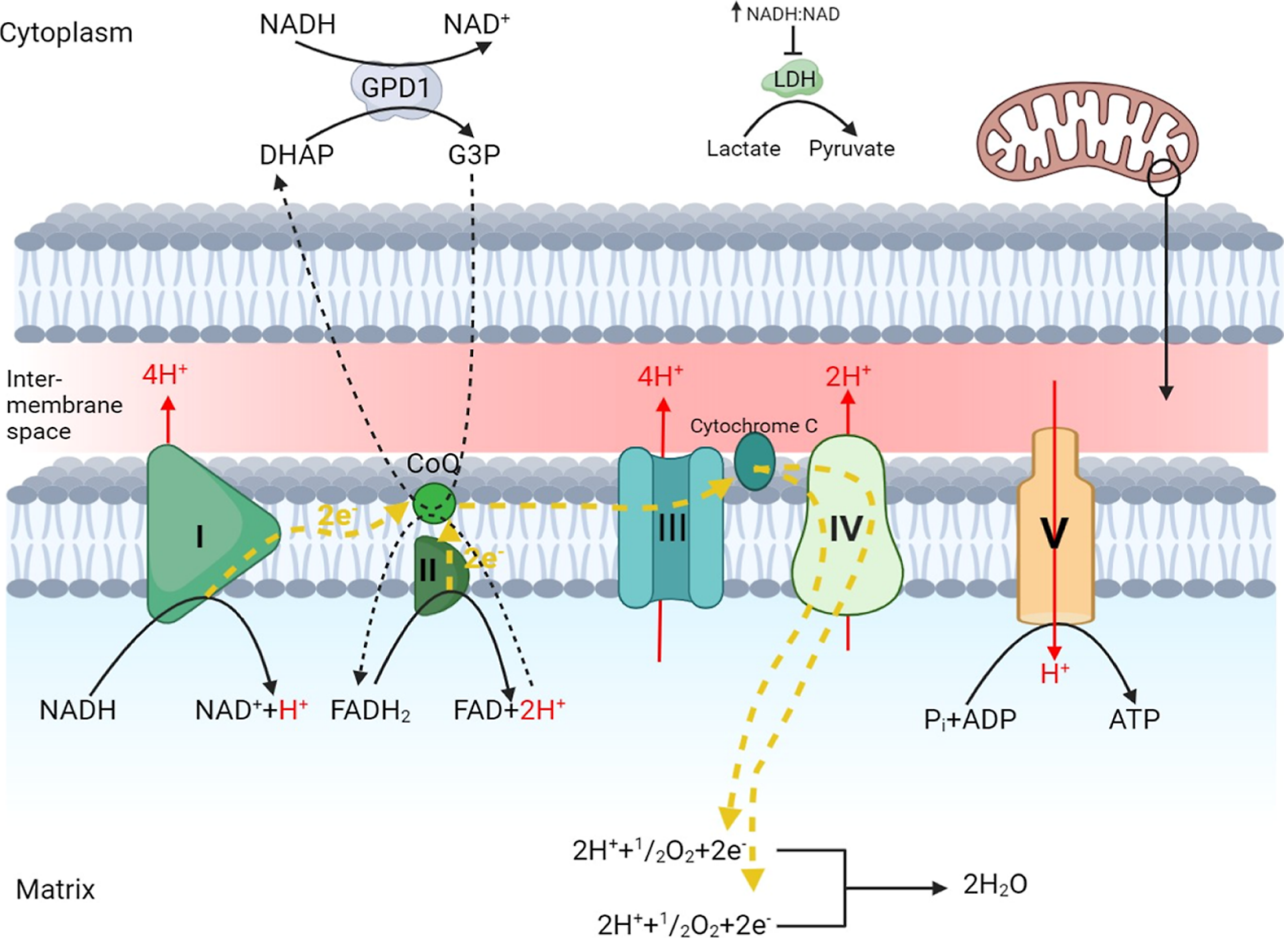
Electron transport chain interacting with GPDH. The process of the electron transport chain occurs in the inner mitochondrial membrane with the intermembrane region responsible for proton concentration. Complex I converts NADH to NAD with liberated hydrogen transferred to the intermembrane space and electrons shuttled via Coenzyme Q10 together, further electrons are liberated from the oxidation of FADH2 through complex III to the cytochrome C, which transfers the electrons into complex IV for reduction to produce H_2_0. The increasingly acidic intermembrane space drives ATP synthase also known as complex V. Outside the mitochondria GPD1 utilizes excess NADH to reduce DHAP into glycerol −3—phosphate while reducing the activity of lactate dehydrogenase. G3P oxidation to form DHAP occurs on the surface of the inner mitochondrial membrane and is driven by an excess of precursors in a process that is reversible in cellular cytosol.

**Figure 4:**
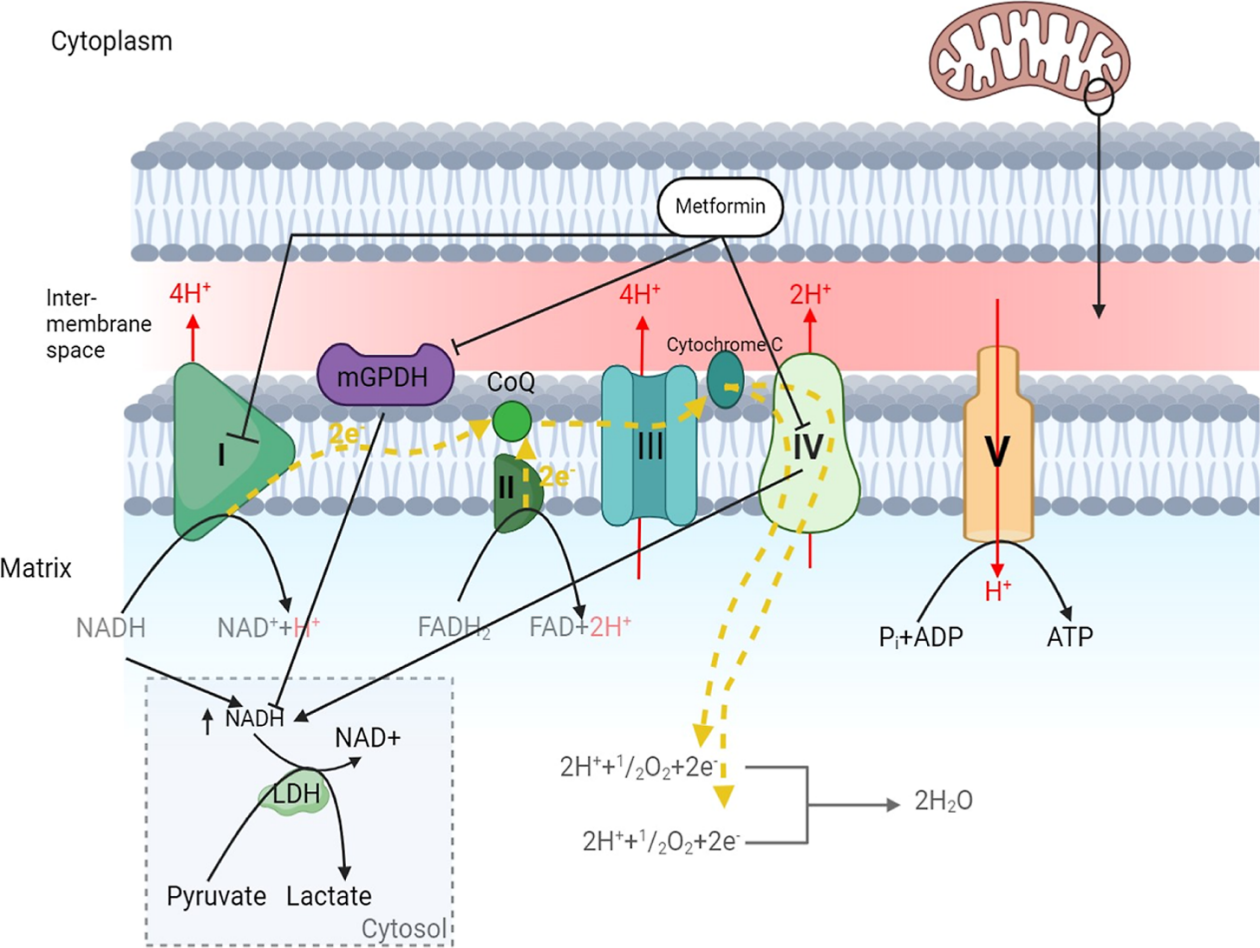
Metformin interacting with the electron transport chain. Metformin has inhibitory activity on complex I and IV resulting in NADH which provides substrates for Lactate dehydrogenase. Inhibitory activities of metformin on mitochondrial glycerol-3-phosphate dehydrogenase reduce the available DHAP that would be used in gluconeogenesis. mGPDH also known as GPD2 is now blocked by metformin, reducing its inhibitory effect on the increase of energetic oxidative substrates driving further reduction of NADH and subsequent lactate production.

Similar to its effect on complex I, metformin acts on mitochondrial complex IV, leading to hypoglycemia and an increased NADH/NAD^+^ ratio, which is further mediated by mitochondrial glycerol-3-phosphate dehydrogenase (mGPDH)^16^ (see Figure 4).

Metformin exhibits a diverse range of effects across logarithmic scales of concentration. Staphenne et al. observed mild AMPK activation at doses of 500 μmol/L and significant inhibition at 1000 μmol/L, with the aim of lowering mitochondrial oxygen consumption rates.^44^ Owen et al. on the other hand, found activity at much lower doses. Metformin concentrations of 50 μmol/L reduced glutamate oxidation and complex I activity, corresponding to plasma levels achieved with oral dosages between 500 mg and 1 g.^38,45^ Evidence is mixed and suggests that supra-pharmacological doses of metformin are often used in vitro to measurably impact mitochondrial complex I.^30,38,40^

## Metformin and mTOR

mTOR is a key regulatory protein that controls cellular growth and is influenced by multiple stimulatory and inhibitory agents. A general overview of the mTOR signaling pathway is available in Figure 1, with Figure 2 outlining the impact of metformin on the system. Belonging to the Phosphoinositide 3-kinases (PI3K) related protein family, mTOR adjusts energy and nutrient equilibrium via various regulatory proteins and drugs.^46,47^ AMPK is a counter-regulatory signaling molecule of mTOR that is activated by AMP in states of energy stress, where the abundance of ATP has been hydrolyzed.^48^ Under normal circumstances, AMPK activation as a result of reducing energy supply results in replication, biogenesis or autophagy, in order to overcome dysfunction within mitochondria.^48^ Outside the mitochondria, AMPK promotes DNA repair through p53 and Caspase 3,^48–50^ and it also has autophagic effects on damaged lysosomes.^48^ Metformin can activate AMPK directly, independent of the AMP-sensitive mechanism by inhibiting v-ATPase.^39^ v-ATPase is an essential inhibitory enzyme required for the function of AMPK and was shown to reduce activity in response to metformin plasma levels of 5 μmol/L, suggesting a direct mechanism for low-dose therapeutic metformin.^39^

AMPK interacting with mTORC1 is involved in regulating various structures, including critical structures involved in myelinated nerve fibers.^51^ Following on from Cameron et al.’s work on cytokine profiles,^52^ Bullón suggests that impaired AMPK function leads to hyperalgesia and is linked to increased levels of the NOD-like receptor family, pyrin domain containing 3 (NLRP3) inflammasome protein.^53^ This elevation in NLRP3 was also observed in fibromyalgia.^53,54^ Pathological interleukin 1β and 18 increases were normalized with Metformin and comparable to a NLRP3 knockout mouse model.^53^ Utilizing a small pilot study, Bullón treated six fibromyalgia patients with 200 mg/day of metformin, noting a reduction in symptoms as measured by the Fatigue Inventory Questionnaire (FIQ) and finding a significant decrease in fatigue.^53^

mTORC1 and mTORC2 are structurally similar signaling complexes with vastly different but occasionally overlapping activities. mTORC1 is activated by an environment rich in nutrients and is responsible for anabolic cellular processes prior to cell division, with increased production of proteins, lipids, nucleotides and amino acids such as glutamine.^55,56^ Inactive mTORC1 is found free floating in the cytoplasm, it is escorted to lysosomes and activated by signaling from organelles and energy precursors such as lipids, glucose and amino acids.^56,57^ mTORC1 is finely tuned and regulated by complex interactions of TSC1/2 (see Figure 1) while its downstream effects have their own counterregulatory effects built in.^56,58^ mTORC1 has several important functions that involve transcription.^56^ These functions include an adaptive metabolism controlled by ATF4 and can also be activated by ER stress. Lysosome biogenesis is another function that is mediated by mTORC1 and inhibited by the transcriptive factor TFEB. mTORC1 also increases lipid synthesis through peroxisome proliferator-activated receptor-γ (PPARγ) and is mediated by SREBP, which can be activated independently by low sterols. Glycolytic activity can also be mediated by mTORC1 through HIF1a, which can be activated independently by hypoxia. Finally, mTORC1 plays a role in mitochondrial biogenesis through PGC1a, which is independently activated by energetic stress. While mTORC1 can be directly deactivated by TSC1/2, there is also indirect inhibition by AMPK which can be increased by metformin.^59^

Unlike mTORC1, mTORC2 is a structurally similar but less acutely rapamycin sensitive protein kinase.^60^ It is involved primarily in cell structure development and survival.^56^ mTORC2 regulates cell structure by affecting the production of actin cytoskeletal structures. Jacinto et al. demonstrated mTORC2 in fibroblasts responding to serum starvation and replenishment by accelerating the output of robust actin filaments.^60^ Akt, in response to insulin and insulin-like growth factors, reduce inhibitory activity toward MTORC1.^61^ However, mTORC1 and mTORC2 can also interact with each other by Akt, which is able to deactivate TSC2 while activating a mSin1, a central component of mTORC2.^56^

Sphingolipids are a major cellular communication molecule on external and internal lipid structures and have been implicated in multiple disease states that have similar clinical symptoms to ME/CFS and LC.^62^ The complex connection between sphingolipids, implicated in several ME/CFS and LC microbiomic and metabolomic studies was demonstrated by Zhang et al.^63–65^ mTORC1 and 2 are major regulators of lipid and sphingolipid synthesis,^56,63,66^ with a decrease in mTORC2 corresponding to peroxisome proliferator-activated receptor alpha (PPAR-α) deficiency and increased lipid droplet accumulation.^63^ The intermediary between the mTORC2:PPAR- α in Zhang et al.’s animal model was demonstrated to be glucosylceramide.^63^ Metformin influencing sphingolipid production and sequestration, modulated by AMPK, has been implicated as the pathway responsible for the beneficial effects of treatment seen in Polycystic Ovarian Syndrome.^67^ Metformin has also been shown to reduce sphingosine-1-phosphate (S1P), resulting in reduced tumorigenesis in ovarian cancer.^68^ The metabolic pathways of sphingolipids^64,69,70^ and glutamine^64,71^ via mTOR and Akt signaling have demonstrated derangement in metabolomic and microbiomic studies of those with ME/CFS and LC, suggesting a possible shared mechanism and offering a potential target for metformin therapy.^72^

Targeting mTOR inhibition via metformin has seen practical therapeutic use in several settings. Shi et al. found reduced AMPK levels as a result of tumor activities within invitro lymphoma samples, resulting in upregulation of mTOR for cellular reproduction.^73^ Focusing therapy toward the AMPK-mTOR interaction (see Figure 2), they demonstrated reduced lymphoma growth both invitro and in xenograft animal models when exposed to temsirolimus and metformin at oral doses of 3 mg/kg/day.^73^ The introduction of Compound C to inhibit all activity of AMPK highlighted the synergistic effect of metformin on AMPK by showing a complete loss of the antiproliferative effect metformin was exhibiting on lymphoma cells.^73^ In addition to the role in cancer pathogenesis, mTOR displays a variety of mechanisms that regulate nervous system functioning. mTOR has significant downstream control of cellular metabolism and neurogenesis via the cytokines stimulated by Ras/MAPK/mTORC1 and also in autophagy of malfunctioning organelles such as mitochondrion.^74^ Of most interest to researchers in neurodegenerative diseases, ME/CFS, and LC, is the direct impact of mTOR on the amyloid plaques seen in Alzheimer’s dementia and neurotransmitter-related enzymes in the brain, such as acetylcholinesterase.^75,76^ The beneficial effect on neurotransmitters is expected to improve the commonly reported cognitive impairment of ME/CFS and LC,^9,13^ while the AMPK inhibition is likely to assist in chronically overactive mTOR as a result of the compensated dysfunctional oxidative phosphorylation pathway.^77^

## ME/CFS

ME/CFS and LC are heterogeneous in presentation and duration,^78^ with several symptoms unique to LC that change and resolve over time.^9^ Heterogeneity, even seen among twin studies and genders complicates the ability to develop a single treatment that will provide unifying symptomatic relief.^79–81^ Trials aimed at treating ME/CFS, such as rituximab, report failures when the diversity in larger populations begins to play a role.^82,83^ We suggest unifying the principle of aggressive symptom management in ME/CFS and LC clustered under affected systems (see Figure 5), whereby whole-of-person treatment should involve targeting of each domain. Although significant interplay between affected domains allows treatment of one domain to improve others, a multifaceted approach is likely to provide greater clinical benefit. Metformin has a strong influence on cellular stress and microbiome derangement (see Figure 6), with medication targeting cardiac autonomic dysfunction in ME/CFS often reporting success in specific subgroups.^84–90^ Similar responses in subgroups are found in psychostimulant medications targeting cognition, suggesting variable penetrance of each domain within individuals.^91,92^ Using the new perspective on treatment, a future strategy might involve autonomic targeting with IV saline,^93,94^ oral rehydration salts^95^ and negative chronotropic medications,^96–98^ coupled with neurocognitive and neuromuscular stimulants.^91,92,99–101^

**Figure 5:**
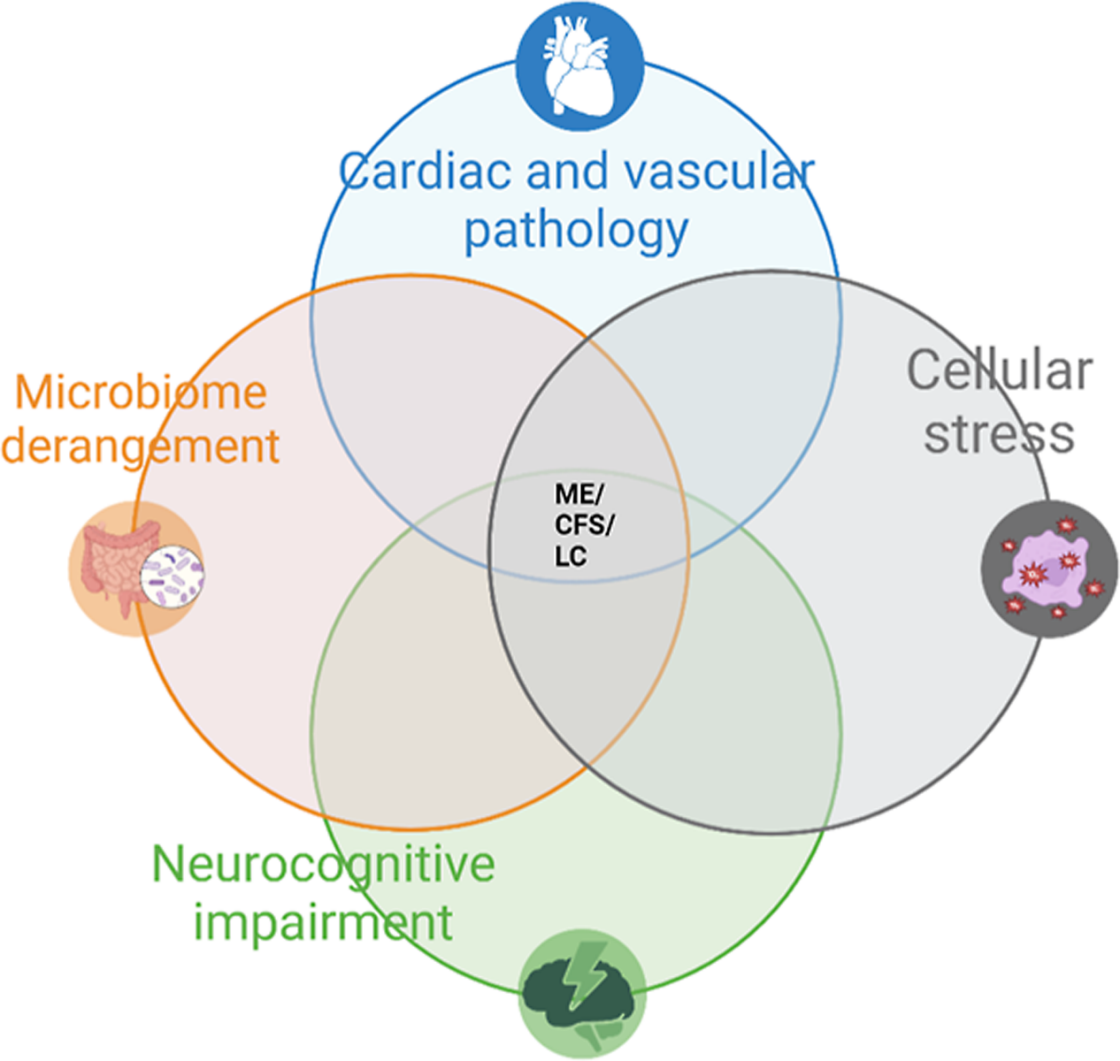
Contributing domains to ME/CFS (myalgic encephalomyelitis/chronic fatigue syndrome) and LC (Long COVID). A simplified diagram demonstrates an overlapping interplay of the etiological pathologies and their contribution to symptomatic ME/CFS and LC. Cardiac and vascular pathology manifests as orthostatic intolerance or breathlessness with signs of postural orthostatic tachycardia and potentially meeting diagnostic criteria for POTS (postural orthostatic tachycardia syndrome). Neurocognitive symptoms such as brain fog and sleep disturbances occur with objective measurable differences in cognitive testing, speech fluency and formal sleep studies. Microbiome derangement is demonstrated by an increase in Irritable Bowel Syndrome and bacterial population shifts. While cellular stress is manifested by post exertional malaise and is evidenced by impaired electron transport chain and free radical generation.

**Figure 6:**
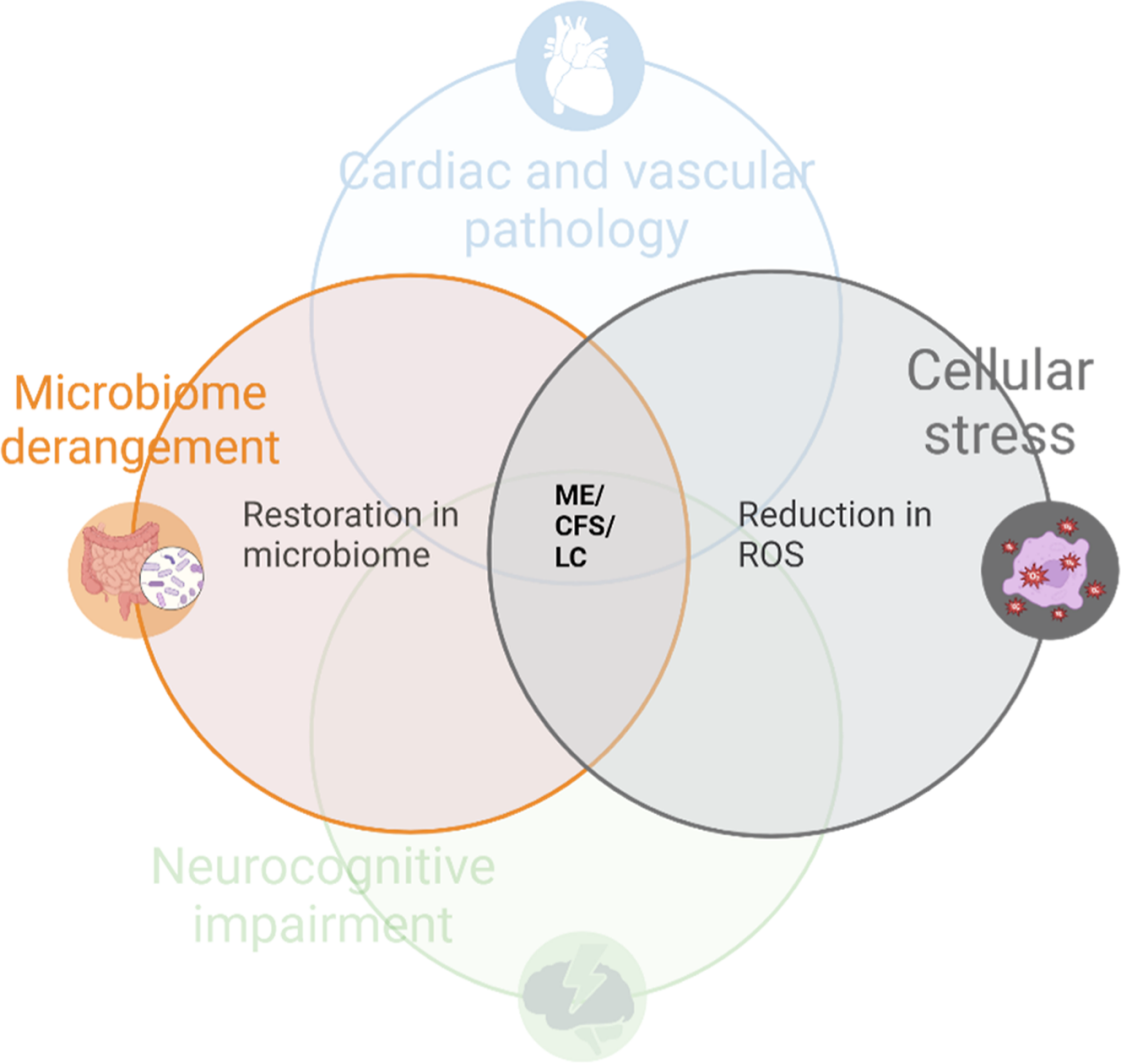
Metformin domain targets in ME/CFS (myalgic encephalomyelitis/chronic fatigue syndrome) and LC (Long COVID). Adapting the diagram from Figure 5, Metformin is seen to reduce ROS (reactive oxidant species) and subsequently reduce the effect of cellular stress on symptoms of ME/CFS. Metformin has a significant influence on the microbiome, in which restoration is likely to reduce symptoms of ME/CFS and LC.

Two groups, Missailidis^77,102,103^ and Wang^104^ made significant headway in the demonstration of pathological underpinnings of ME/CFS that offer new guidance into potential medical intervention targeting the cellular stress domain. In 2020 Missailidis et al. established several critical findings in biomarker analysis of ME/CFS that expanded understanding and opportunities for therapeutic intervention.^77^ PBMC survival following long-term frozen storage was dramatically reduced in ME/CFS patients compared with healthy controls and possessed significant abnormalities in the signaling pathways of mTORC1.^77,102^ Lymphoblasts from 51 ME/CFS patients were analyzed by Seahorse Extracellular Flux Analysis to measure oxygen consumption rate together with ATP assays. These experiments found a significant reduction in ATP synthesis in complex V of ME/CFS patients, while unexpectedly demonstrating elevated O_2_ consumption in upstream mitochondrial complexes.^77^ This suggests intra and extramitochondrial recruitment of compensatory mechanisms to sustain energy production required for normal cellular processes.^104^ The difference in oxygen consumption across complex V was 15% less than healthy controls. Proteomic analysis showed an increase in the transcription of components of complex V suggesting a hypofunctioning protein was the cause of reduced metabolic rate.^77^ The failure of the electron transport chain at complex V, normally responsible for utilizing electrons to produce ATP, instead results in the hypermetabolic creation of free radicals,^81,105^ that if not adequately buffered, result in damaging oxidation of cellular structures.^81,106^

Mitochondrial membrane permeability allows a flow of toxic acidic substrates into the local environment, with significant microclimatic effects and is susceptible to therapeutic metformin. The reduction in proton leak is crucial in maintaining an efficient energy supply for cellular function, while a certain amount of physiological fluctuation is useful as a messaging molecule.^107^ Missailidis et al. found a proton leak in mitochondria that reduced transmembrane charge and induced mTORC1 hyperactivity.^77^ This was measured by a significant increase in phosphorylation of an mTOR substrate, 4 EPB-1.^77^ All these alterations occurred without a change in ATP levels, suggesting the engagement of extramitochondrial compensatory mechanisms.^77,108^

Evidence of an impaired electron transport chain resulting in patterns of biomarkers and symptoms of ME/CFS was established by Missalidis et al.^77^ and Keller et al.^109^ Accepting this, the question remains: What is the interfering agent? In vitro and animal studies of the protein WASF3 showed that an increase and knockdown had demonstrable effect on complex IV activity, with typical upstream backflow.^104^ Wang et al. focused on localizing the source of dysfunction in cells overproducing WASF3, determining the interaction was post translational, with higher mRNA but lower protein production of complex IV.^104^ In addition, they further demonstrated that upstream upregulation of compensatory mitochondrial respiration mechanisms were mediated by p38, a signaling pathway commonly activated by ROS, hyperosmolarity, bacterial pathogens and cytokines.^104,110^ Measuring oxygen consumption and extracellular acidification lead to findings of increased glycolysis and lactate production,^104^ a common phenomenon in ME/CFS.^111–113^

The endoplasmic reticulum (ER) is a metformin-sensitive organelle comprising of folded lipid membranes serving as storage and production centers of various ions, molecules and proteins. Wang et al. suspected that ER stress and its regulation was implicated in WASF3 toxicity.^104^ They measured the levels of ER regulator proteins BiP and PERK (see Figure 7), unexpectedly finding an overabundance of PERK with a reduced BiP.^104^ This suggests either BiP had been overcome by its primary quality control role for protein folding or had been inappropriately relocated to the cell surface. Animal models tested the results of measuring the response to ER stress induced by lipopolysaccharide injections showing an increase in WASF3, PERK and BiP, with a reduction in mitochondrial complex IV subunits.^104^ Wang et al. found an unanticipated discordance in activated PERK and its downstream activated protein eIF2a, suggesting an error in the ER stress signaling system.^104^ Similar findings have been found in traumatic brain injuries that can result in similar symptoms to ME/CFS.^114,115^ While Wang et al. utilized a very small sample, the findings exhibit a novel mechanistic logic in the disease process of ME/CFS. Metformin has been shown to reduce ER stress in addition to reductions in mitochondrial stress.^116,117^ Metformin affecting ER stress results in reduced downstream eIF2a phosphorylation in vivo at therapeutic, albeit high doses.^117^ Persistent pathological dephosphorylating activity extending beyond the acute infection^118^ is a plausible explanation of the post viral onset of ME/CFS and LC that may be ameliorated with metformin.

**Figure 7:**
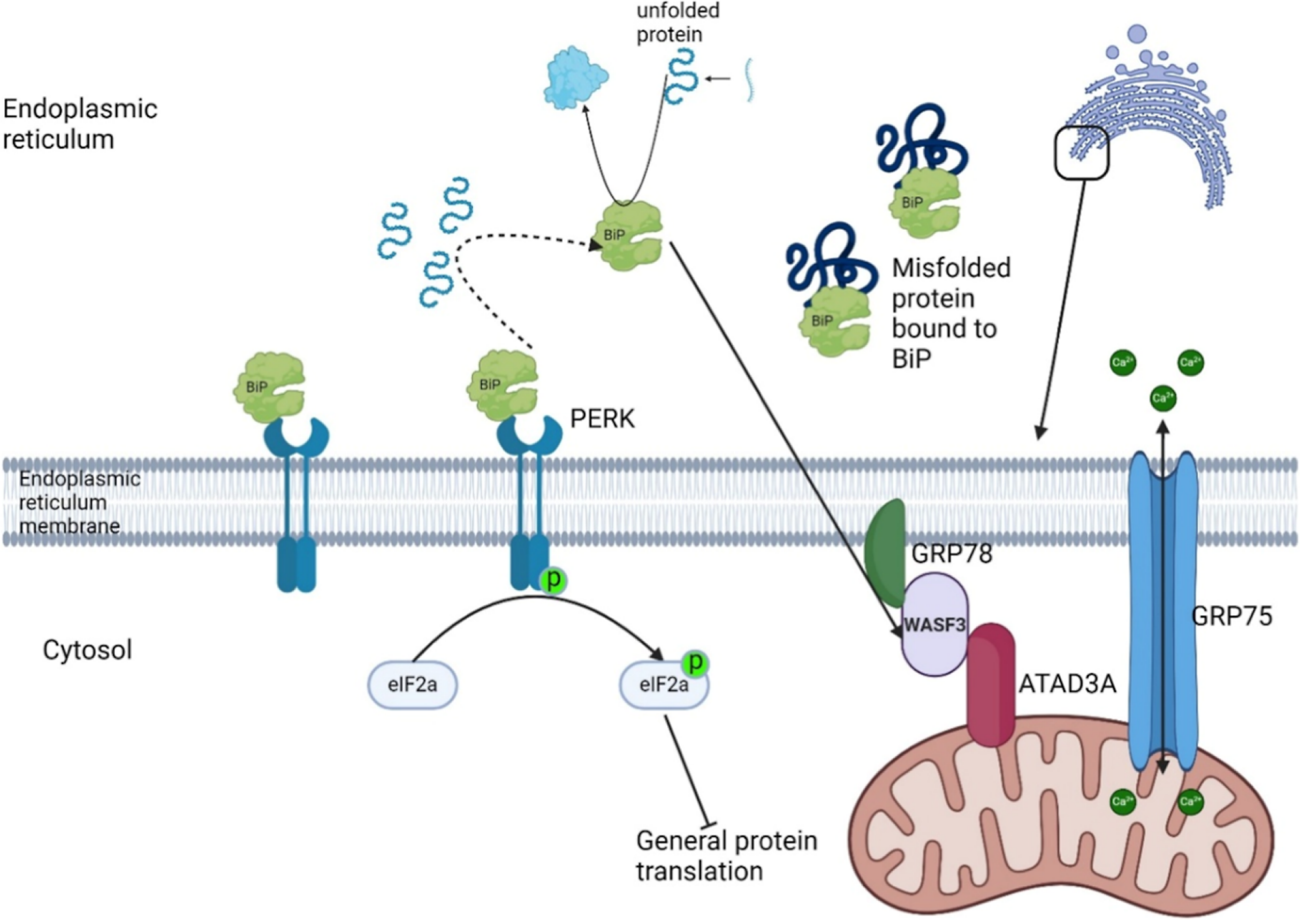
The role of WASF3 in the cellular microclimate. The mitochondria are linked to the endoplasmic reticulum (ER) with a complex, comprising of WASF3 between GRP78 on the ER side and ADAD3A on the mitochondrial side. GRP75 transmembrane channel allows for calcium transmission between mitochondria and the ER. BiP dissociates from and activates PERK due to BiP’s attraction to unfolded proteins. BiP functions to assess the fold of a protein ensuring they have achieved the correct shape. BiP also functions to chaperone proteins such as WASF3 to their intended locations. An excess of unfolded proteins (ER stress) results in more DiP dissociation from PERK which autophosphorylate and activate eIF2a, reducing general protein production to allow cellular mechanisms to catch up. Misfolded proteins reversibly bind BiP and accumulate in the ER lumen, preventing reattachment to PERK.

Beyond interactions with biochemical pathways exists a local microclimate of the mitochondria, endoplasmic reticulum and nucleus.^119^ Sharing of calcium via GRP-75 was found to occur through trans organelle channels.^120^ Alterations to interacting proteins on both ER and mitochondria in response to ER stress from hyperglycaemia were shown to affect calcium homeostasis with an initial increase in proximity, followed by dysfunction.^120,121^ The effect was reversed with metformin but not rosiglitazone, an antihyperglycaemic agent of differing methods of action.^122^ This suggests that metformin’s direct actions and not simple glucose reduction is involved in mitochondrial and ER structural vicinity.^120^ Extrapolating these findings together with Wang et al.’s WASF3 study suggests a shared mechanism of disease that may be therapeutically ameliorated with metformin.^104^

## Glutamine, Glutamate, Glutathione and ME/CFS

Glutamine and its metabolic products function as abundant neurotransmitters and antioxidants, however when required can be utilized as an alternative energy source. Metformin can modulate glutamate and downstream metabolites through several different mechanisms. Alterations in glutamic metabolites have been recognized in biochemical and neuroimaging studies on those with ME/CFS. Renz-Polster et al. retrospectively examined the neuroimaging cases of Gay et al.^123^ and Shan et al.^124^ finding elevated glutamate levels.^125^ A series of neuroimaging studies published from 2009–2012 tested the presence of cerebral lactate and glutathione, a marker of oxidative stress in ME/CFS patients, against those with depression and anxiety,^71,112,113^ finding elevations only within the ME/CFS population. Unfortunately, it was not until 2015 that technology had advanced enough to separate the overlapping glutamine/glutamate/glutathione signals.^126^

More advanced neuroimaging and spectroscopic studies eventually demonstrated this altered metabolic profile. Godlewska et al. recruited 22 ME/CFS and 13 healthy controls for neurometabolic studies using a high-powered 7 T scanner.^127^ They demonstrated a reduction in creatine, glutathione, and myo-inositol to a level of significance, with a minor drop in glutamate (9.19 (0.21), 9.93 (0.34) *p* = 0.052) that did not meet significance due to population size, without a measured change in glutamine.^127^ Glutathione is a downstream product of glutamate redox reactions and is critical to maintaining homeostasis of ROS.^128,129^ Utilizing this pathway would suggest metabolic shunting from glutamate away from its usual redox reaction into alpha-ketoglutarate to be metabolized as part of the TCA cycle by the enzyme glutamate dehydrogenase,^130,131^ which ADP positively modulates.^132,133^ Functioning as a preferential antioxidant within mitochondria, glutathione is consumed as ROS increases during the dysfunctional metabolic processes (see Figure 8).^128,134,135^

**Figure 8:**
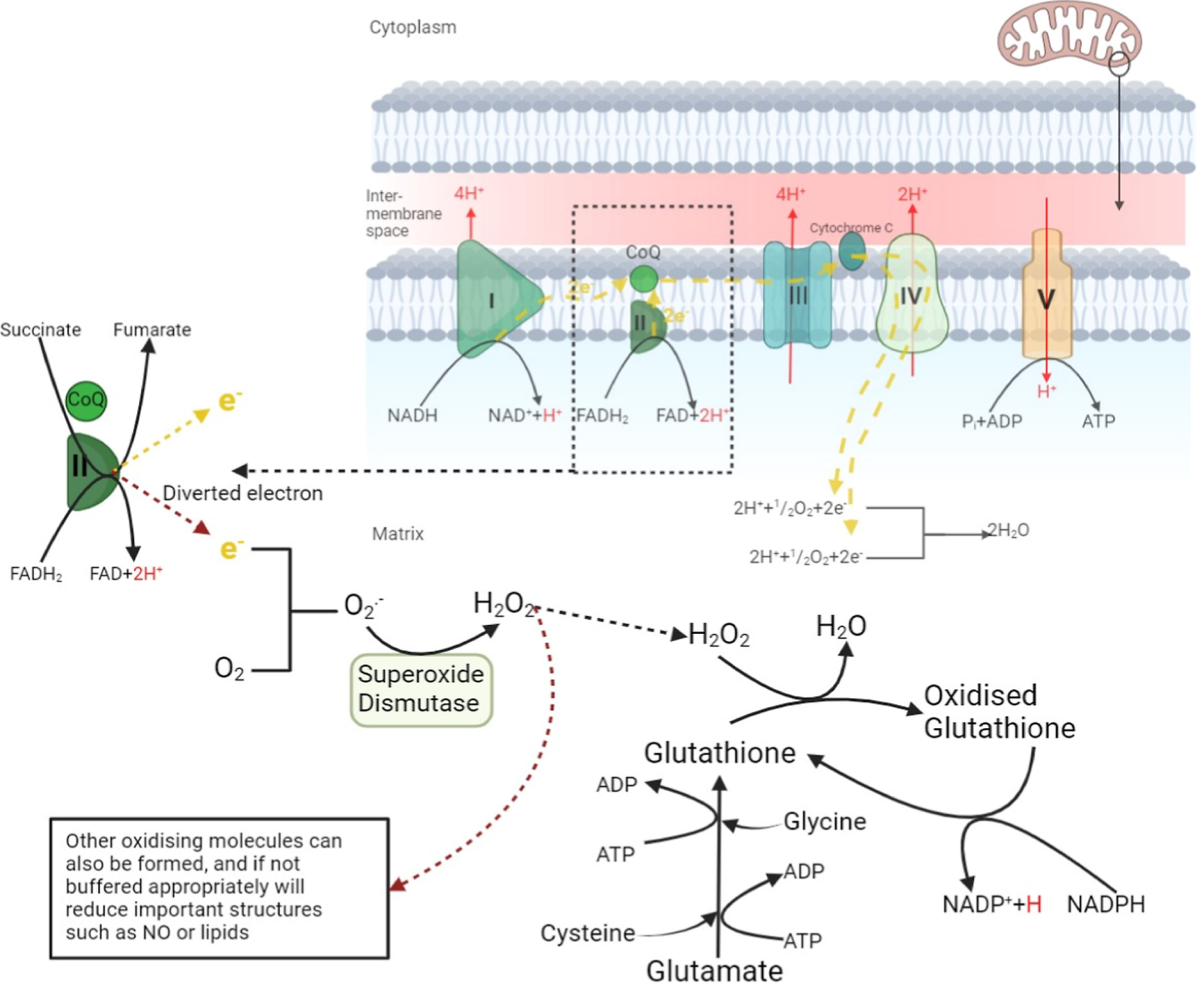
Mitochondrial ROS generation. Mitochondrial reactive oxidant species are formed from aberrant electrons. These electrons can interact with a variety of cellular structures as the mitochondria attempt to reduce membrane charge. In this example hydrogen peroxide is formed by the enzyme superoxide dismutase. Glutathione is seen buffering the oxidation in a potentially reversible reaction. Cells with increased ROS formation require an abundance of buffering agents. Glutamate is freely available and is irreversibly consumed to provide this buffer.

The shift toward anaerobic respiration produces more lactate and less ATP per glucose than aerobic respiration. Cells require a minimum amount of ATP to maintain integrity of critical cellular functions. Magnetic Resonance Spectroscopy has the capacity to demonstrate a reduced glutathione as its precursor glutamate is shunted into the TCA to produce the necessary energy. Engagement of these compensatory mechanisms, resulting in elevated lactate to maintain cellular ATP production was demonstrated by Godlewska et al.’s neuroimaging studies.^127^ Improving on these findings were in vitro experiments performed by Islam and Manna showing supratherapeutic metformin reducing the cellular buffering demand to ROS.^136^ They demonstrated stable antioxidant abundance of glutathione, reduced glutamine transport activity and reduced cysteine/glutamine ratio.^136^ Glutamine is abundant in the brain, and the simple transition between glutamine and glutamate is expected to rapidly achieve stability with the available resources. The sources that these amino acids are drawn from, however, are sensitive to fluctuation and present a plausible explanation for the symptoms of ME/CFS. Specifically, within the paraventricular nucleus, glutamate is the predominant neurotransmitter influencing blood pressure as well as sympathetic tone and wakefulness.^130,137,138^

In addition to the compensatory mechanism is the potential ability of metformin to reduce ROS by improving efficiency of mitochondrial complexes. Mitochondria in response to increased fatty acid oxidation have been shown to bunch together complexes I–IV resulting in a “super-complex”, manifesting increased efficiency and reduced ROS generation.^139^ Metformin has been shown to increase the availability of these fatty acid precursors while reducing antioxidant consumption.^136^ While palmitic and other fatty acids are often seen upregulated in ME/CFS they are usually accompanied by ROS generation.^140^ It is possible that the supercomplex formation is either a result of a decoupled pathological complex V,^139^ or a compensatory mechanism induced by mitochondria to reduce oxidative stress.

ME/CFS is a multisystem disorder and demonstrates similar disturbed metabolic processes in peripheral tissues. Armstrong et al. recruited 46 ME/CFS and 26 healthy controls to perform metabolomic Nuclear Magnetic Resonance (NMR) analyses on blood and urine samples, demonstrating a paucity of glutamate with a disproportionally elevated glucose.^141^ Similar deranged mitochondrial metabolic pathways have been confirmed by Holden et al.’s systematic review, suggesting that ME/CFS is a metabolic mitochondrial illness.^142^ This defective metabolic pathway results in reduced glutaminergic neurotransmission in the paraventricular nervous system with associated autonomic symptoms and fatigue, providing a causal etiological link. Shankar et al. demonstrated similar cell-based pathology in ME/CFS and LC finding a significant elevation in markers of oxidative stress over that of healthy controls.^81^ Surprisingly, results showed that males did not exhibit similar ROS elevations to female counterparts, with downstream oxidized cellular substrates in males being more likely to exhibit higher lipid peroxidation, while females demonstrated elevated mitochondrial calcium and higher hydrogen peroxide.^81^ Higher GSH (reduced glutathione) and reduced superoxide dismutase were seen in both sexes.^81^ These findings show a chronically upregulated glutaminergic antioxidant pathway, extended beyond capacity to accommodate transient spikes in oxidative stress, which are typically generated by exertion. This lends support to the hypothetical link between the role of glutamate fluctuation in PEM. Following the identification of affected pathways, Shankar et al. attempted to reduce the oxidative stress by treating in vitro T cells with 10 μmol/L metformin for 5 days, finding that ME/CFS cells demonstrated a reduction in markers of ROS while there was no change in healthy controls.^81^ These findings, together with similar reductions in ROS observed after *N*-acetyl-cysteine administration in vivo and in vitro, highlight an inadequately activated glutamatergic antioxidant defense mechanim.^81,143^

The link between pathological actions of glutamate as a neurotransmitter and oxidative stress buffer in ME/CFS can be influenced by metformin and is suspected to involve Methylglyoxal (MG). MG is a metabolic byproduct of DHAP during glycolysis that requires glutathione to convert into d-lactate.^144^ d-Lactate as a biomarker of disease, has been found in ME/CFS and exhibits deleterious glycosylating effects on cell structures contributing to the vascular complications of diabetes.^145–147^ Vizuete et al. demonstrated profoundly reduced hippocampal glutamate uptake in astrocytes exposed to MG, further demonstrating the role of metformin in increasing glutamate while reversing inhibitory activities of MG.^148^ NMDAR1 is a glutamate-gated calcium channel that has increased expression in response to MG exposure and is reversible by metformin.^148^ NMDAR1 upregulation is expected to occur as a result of glutamate starvation induced by MG in order to maintain homeostatic downstream signaling. Vizuete et al. demonstrated metformin phosphorylating glutamate transporters resulting in structural changes that boost resilience to damage from MG glycation.^148^ Vizuete et al. showed metformin reducing IL-1β, and built on earlier findings from Cameron et al. by establishing the interference of metformin with MG-derived advanced glycation end products and their receptors (RAGE).^52,148^ RAGE activates inflammatory protein synthesis via NFκB and is mediated by NLRP3.^53^ Vizuete et al. demonstrated that neuronal cells have the capacity to upregulate their NMDA receptors rapidly.^148^ This might be seen in PEM-triggered glutamate depletion with glycolytic shifts. Subsequent glutamate toxicity resulting in neuronal injury may be an explanation for the persistent cognitive impairment in those with ME/CFS and LC. The ability of metformin to regulate glutamic flux has the potential to heighten the threshold for PEM while reducing resultant neuropathic damage.

## Anti-Inflammatory Actions of Metformin

Cytokines are chemical messengers that are released as part of an inflammatory or infective response. Disease states can result in overexpression of cytokines and have been targeted by several drugs to induce therapeutic responses. Metformin displays anti-inflammatory effects through interactions with leucocytes and the various cytokines. Cameron et al. studied the in vitro anti-inflammatory effects of Metformin, finding inhibition of TNF-α-induced IκB degradation and reduced expression of the proinflammatory cytokines IL6, IL-1β and CXCL1/2.^52^ The experiment determined that 3 h of Metformin exposure at 2 mmol/L was adequate to activate AMPK and suppress TNF-α induced inflammatory signaling.^52^ The immunological response to metformin normalizes inflammatory cytokines and presents a measurable mechanism for the treatment of ME/CFS and LC.

Neutrophils are the most abundant leucocyte in the blood and together with lymphocytes are responsible for the vast majority of immune actions of the body. Cameron et al. built on data from their in vitro study by undertaking a prospective cohort trial on people with T2DM who were provided with metformin (*n* = 498) or sulphonylureas (*n* = 172), measuring a reduction in the neutrophil to lymphocyte ratio (NLR) of 9% (2–17 *p* = 0.01).^52^ This demonstrates metformin’s influence on the NLR has a substantial impact on inflammation and adaptive immunity.^52,149^ The NLR had seen significant utility in the triage of severe cases of acute COVID-19 during the early phases of the pandemic,^150^ as well as demonstrating correlations to mood disorders and geriatric postoperative fatigue.^151,152^ Unlike their cohort trial, Cameron et al. used data from a randomized placebo-controlled trial to test the effect of Metformin on the cytokine levels.^19,52^ They recruited 20 participants 4 months from starting Metformin, together with 12 controls showing a highly selective reduction in the proinflammatory cytokines CCL22 and stromal cell derived factor 1αβ.^19,52^ Macrophages use oxidative markers to limit runaway inflammatory responses through GPD2 and presents another metformin sensitive target for treatment.^16,153^

Supplementing its anti-inflammatory properties, Metformin has potential direct antiviral properties. Chen et al.’s review discusses Metformin’s role in AMPK-mediated inhibition of viral replication.^23^ Viruses such as Dengue and Coxsackie B3 required a considerable concentration of 1 mmol/L metformin to achieve in vitro antiviral activity.^154,155^ Similar concentrations of metformin were used to modulate AMPK and reduce HHV8 (Kaposi Sarcoma associated Herpesvirus) lytic replication despite enhanced latent RNA transcription.^24^ The proposed antiviral mechanism of AMPK suggests avoidance of HMG-CoA reductase (HMGCR) phosphorylation, which reduces its activity. This increase in active HMGCR reduces cholesterol accumulation on the endoplasmic reticulum of infected cells, limiting the capacity of lipid biosynthesis required for viral replication.^154,155^ Linking antiviral activities of metformin with antineoplastic effects, Islam et al. found elevated hypoxanthine, a precursor for denovo purine synthesis, and reduced adenine in response to 2.5 mM metformin.^136^ The evidence of post viral ME/CFS,^3,156,157^ LC onset,^2,158^ and shared symptoms of fatigue and brain fog experienced by those undergoing chemotherapy^159^ suggest a commonality that may be exploited by a reduction in underlying proliferative capacity.

## Metformin for MCAS (Mast Cell Activation Syndrome)

MCAS refers to a condition involving the stimulation of mast cells via toll-like receptors to release their contents containing chemokines, cytokines, histamine, proteases and prostaglandins.^160^ In their most severe form, these products cause anaphylaxis; however in MCAS, they produce symptoms commonly manifesting as gastrointestinal disturbance, cutaneous symptoms, nasal congestion and itch, throat swelling, wheezing, angioedema, headaches and cognitive impairment with fatigue.^160,161^ Diagnostic criteria have been suggested to involve typical clinical symptoms associated with an increase in serum tryptase level and an improvement in response to therapeutic histamine blockade.^161^ MCAS is a common condition comorbid with ME/CFS and LC, with many shared biochemical and immunological mechanisms susceptible to influence by metformin. Reviewing the differing actions of Metformin, Ibrahim et al. suggest that in addition to MTOR, AMPK, immunomodulation, MAC and PTP mediation, that other potential mechanisms may be involved, such as mast cell stabilization, reduced thrombotic risk, reversal of established fibrosis and alterations in endosomal pH.^21^ There is significant crossover between symptoms and inflammatory mediators between MCAS and ME/CFS.^84^ MCAS has a baseline prevalence of 17% of the population, with family members 2.7 times more likely to suffer the condition.^162^ Those affected experience a significant reduction in the quality of life, with fatigue, like ME/CFS and LC, the most prevalent symptom.^163^ Host responses to viral infections frequently involve an element of mast cell activity in addition to the humoral and cellular mechanisms of defense. Mast Cell degranulation resulting in inflammation was shown to be triggered by COVID-19 through viral inhibition of Interferon and Nuclear Factor-κB.^164^ The accumulated immunomodulatory mechanisms are further discussed in Ibrahim et al.’s review,^21^ which support Wang and Huang’s position on the immunomodulatory activities of Metformin.^69^ Wang et al. demonstrated ligand stimulation of Aryl Hydrocarbon Receptor (AHR), mediated by SHP-2 phosphatase produced ROS and liberated calcium ions from the endoplasmic reticulum in mast cells.^121^ Calcium ions were not released via rupture of the ER membranes, but by passive leak following interference of ROS-sensitive Ca^2+^ ATPase proteins.^121^ The AHR stimulated calcium depletion increases mitochondrial ROS and Ca^2+^ toxicity, resulting in loss of membrane potential and cell death.^121^ Following the connection between AHR and pathological mitochondrial and ER changes, Wang and Huang found that metformin suppressed FcεR1-mediated degranulation, IL-13, TNF-α and sphingosine-1-phosphate (S1P) secretion despite a provoked AHR release.^69^ As S1P extracellular and intravascular concentration increases in response to inflammation, renal clearance of histamine is enhanced. This reduction in circulating histamine functions to counter histamine’s vasoactive effects, reducing permeability and increasing vessel tone.^165^

Displaying logarithmically increasing doses for different effects is a key feature of in vitro studies of metformin. While supratherapeutic levels have often been demonstrated to achieve significant in vitro results, mast cells respond to much lower doses. Metformin concentrations required to achieve mast cell stabilizing effect were within 1–10 μmol/L and achievable through an oral administration of 500 mg daily.^45,69^ Levels of 1 μmol/L showed a significant reduction in IL13 and S1P but required concentrations of 10 μmol/L to achieve significance against TNF-α.^69^ Given Metformin’s ability to reduce degranulation, it is expected to influence the MCAS traits with subsequent reduction in the most common symptom of fatigue. Whether this treats the comorbidity of MCAS in ME/CFS or has an overall effect on fatigue will require further investigation.

## Metformin and COVID-19

Early COVID-19 research focused on retrospective analyses to help direct future investigative trials into therapeutics. An extensive retrospective cohort control analysis from Hubei province in China assessed the impact of COVID-19 on people with type 2 diabetes taking metformin.^166^ The nonmetformin Cohort demonstrated higher cerebral and coronary vascular events; however, unexpectedly worse glycaemic control was displayed in the metformin group despite rigorous methodological adjustments.^166^ The metformin group displayed a higher rate of acidosis and lactic acidosis (2.95%, 1.77% vs 1.5%, 0.75% respectively), however a lower rate of heart failure (HR 0.61 *p* = 0.006) and Acute Respiratory Distress Syndrome (ARDS) (HR 0.66 *p* = 0.028) with no significant difference in mortality between groups.^166^

Later in the pandemic, novel and impactful adaptive platform trials emerged with highly translatable findings. Bramante et al. published the results of multiarm, multiagent, parallel-grouped, phase three trials targeting preventative agents for LC.^27^ Participants with acute COVID were assigned on equal allocation to treatment arms of metformin plus ivermectin, metformin plus fluvoxamine, metformin plus placebo, ivermectin plus placebo, fluvoxamine plus placebo, or placebo only. Confirmed cases of LC by day 300 was the primary end point, with a total of 8.3% of the initial n of 6602. Both ivermectin and fluvoxamine had no impact on incidence, but the metformin group had positive results, showing a reduction of 41% when instituted within 3 days of the onset of COVID-19 symptoms.^27^ A rapid increase in the dose of metformin was utilized by Bramante’s COVID-OUT team using an instant release Metformin preparation of 500 mg daily on day 1 of the 500 mg BD days 2–5 the 500 mg morning 1 g evening for a total of 2 weeks therapy.^27^

When applying these trial findings to local clinical cohorts, attention should be directed toward the differing primary course vaccination rate among the included population and circulating viral strain. Of 1126 participants in the US, only 55% had completed a primary course of SARS-CoV-2 vaccination.^27^ This vaccination rate vastly differs from Australia, which reports only 315,000 unvaccinated eligible individuals in the country from a report in early January 2024.^167^ The contrast between the 55% trial unvaccinated and the 1.16% unvaccinated population suggests that data may need local comparison before implementation. Similar differences in completion of primary vaccination course are seen globally, with over 79% of individuals in the US reported on May 2023 by the CDC with a current global vaccination rate above 64.9%.^168,169^ The disparity in groups was accounted for during analysis, with similar benefits conferred to both the vaccinated and unvaccinated groups. This benefit is suggested to be additive to the reduced rate of Long Covid seen in vaccinated individuals.^170^

Reducing virulence and compounding infections are likely to impact the emergence of LC in unpredictable ways. COVID-OUT recruited participants between the end of 2020 and the start of 2022, where the prevalent strains of COVID-19 comprised of 89% Omicron BA1-3 and 10% Delta, with small numbers of Alpha, Beta, and Gamma.^27,171^ The Omicron variant JN.1 at the time of writing was responsible for a rising prevalence in over 88% of cases in the middle of February of 2024, with an ongoing reduction in virulence seen by a falling ICU admission-to-hospitalisation rate from its peak in July 2021, which corresponded to a measurable decrease in mortality.^172^ This would suggest that the emergence of newer variants are selecting for more infectious but less acutely severe characteristics, resulting in a lower incidence of LC.^170^

Before Bramante et al.’s comprehensive trial,^27^ Reis et al. used an adaptive platform trial design to test multiple drugs, one of which included metformin to assess the impact on acute COVID-19 infections.^173^ Using a population at elevated risk of severe COVID-related illness, they administered 750 mg bd Metformin or placebo for 10 days, commencing early on in the acute phase of infection. Primary outcomes were set at residing in any hospital unit for longer than 6 h and a secondary end point of viral clearance at day 7.^173^ The nature of adaptive platform trials requires establishment of a “minimal clinical utility” in the form of risk reduction to help guide the promotion or abandonment of the trial medication. Reis et al. set this efficacy rate at 37.5% with an established control event rate of 15%, aiming to terminate early if measured outcomes fell below 40% or above 97.6%.^173^ Compared with the placebo, there was a relative risk of 1.14 (0.73–1.81) of metformin worsening the primary outcome, resulting in early termination of the trial for futility.^173^ While this population had significant comorbidities, there was no benefit in the hospitalisation rate compared with the control. A significant difference in adherence was noted, with 21.9% of the Metformin group dropping out compared to 11.8% of the placebo group, underscoring the difficulty of administering metformin during the acute stages of a COVID-19 infection.^173^

Later studies confirmed the unimpactful response of metformin on duration of hospitalisation, however unlike Reis et al.’s TOGETHER trial^173,174^ Ventura-Lopez et al.’s randomized controlled trial found a reduction in severity of acute COVID-19 infection and reduced viral load in response to 620 mg Metformin Glycinate twice daily.^175^ Ventura-Lopez et al. ran a parallel in vitro study and determined that Metformin Glycinate required 189 μmol/L for cell-associated IC_50_ on viral load, and supernatant viral load required 355 μmol/L to achieve IC_50_.^175^ The accumulating evidence suggests that metformin has some role in treating COVID-19. However, the changing landscape modulated by variant emergence, antiviral use, and vaccination rates will require time and updated studies before we are able to determine the risk–benefit profile and subsequent guideline implementation.

## Metformin’s Vascular Effects

Metformin has been shown to affect vascular morbidity and mortality, reducing cardiovascular death and coronary events independent of the presence of diabetes.^176^ Vascular endothelium has significant cardioactive paracrine functions, releasing natriuretic peptides, prostanoids, and nitric oxide, as well as expressing ACE.^177^ Vascular endothelial damage had been noted in many early LC trials that found extensive changes in nailfold capillaroscopy.^178–180^ ME/CFS exhibits similar endothelial dysfunction, with Sandvik et al. measuring impaired reactive hyperaemia and flow-mediated dilation of the brachial artery diameter in response to transient occlusion and administration of GTN.^181^ The return to normal baseline from a pathological state following exogenous GTN administration suggests impaired endothelial baseline nitric oxide (NO) level. This difference in baseline endothelial dysfunction as measured by reactive hyperaemic index was quantifiably reduced from healthy controls (1.95 ± 0.47 vs 2.21 ± 0.37, *P* < 0.05) and correlated to fatigue severity in ME/CFS.^182^

Nafisa et al.’s comprehensive review outlines the various agents acting and being produced by vascular endothelium and discusses the action of metformin in these domains.^183^ They suggest that metformin’s reduction of the endothelium-specific micro RNA miR34a and direct enhancement of GTPCH1 affect vascular NO availability and subsequently improve vascular responsivity, despite underlying endothelial dysfunction.^183^

## Metformin as a Neuroprotective Agent

Metformin has the ability to protect neuronal cells from death by reducing the efficiency of the Permeability Transition Pore (PTP) in mitochondria.^184^ When Mitochondrial Apoptosis-induced Channel (MAC) is activated, cytochrome *c* leaks from within the mitochondrion, forming apoptotic protein complexes.^185^ At the same time, PTP results in permeability change of the inner mitochondrial membrane, with fluid shifts resulting in matrix expansion and necrotic breakdown of the outer membrane.^186^ Interestingly, in this same article, Propranolol is noted to block both PTP and MAC at 700 μmol/L and 52 μmol/L respectively.^186^ Morin et al. suggest the possibility of utilizing tricyclic antidepressants and the less tolerated Cyclosporin A as protection against ischemia-induced mitochondrial hyperpermeability.^187^ Propranolol and tricyclic antidepressants are frequently found within the practitioner’s toolkit for treating ME/CFS and LC, suggesting a commonality among potential etiologies.

## Metformin and the Gastrointestinal System

The interplay between commensal organisms colonising the human body and their effects on human physiology is an emerging field of study. Owing to gastrointestinal side effects and the fundamental methods of action of metformin, the involvement of GI bacteria is suggested to be an intended or unintended drug target. Buse et al. utilized a delayed absorption drug delivery design to target the increased gut concentration abilities of metformin in the small intestines in an attempt to increase ileal exposure and limit extra-intestinal uptake.^188^ Despite the low plasma metformin level, they found significant glycaemic reduction where 1000 mg delayed release metformin corresponded to 70% of the effect of 2000 mg sustained release preparation.^188^ These findings suggest that local intestinal actions of metformin exert a significant proportion of the effect compared with downstream hepato-systemic regions.

Metformin has been shown to alter intestinal bacterial composition in healthy people and those with T2DM. 1000 mg of metformin was given twice a day to 23 healthy volunteers, finding a change in 11 species that returned to baseline following cessation of treatment, with several species fluctuating during the first 3 weeks of therapy.^189^ Both Bryrup et al. and Elbere et al. demonstrated similar findings in assessing microbiota changes to metformin, finding a reduction in *Clostridiacaea* and an increase in *Escherichia/Shigella*.^190^ Wu et al. echoed similar findings through a six month longitudinal trial, confirming a persistent reduction in small chain fatty acid producing organisms through pathway enrichment analysis.^191^

Histamine is a central gastrointestinal signaling molecule with subtyped receptors producing differing roles, ranging from intestinal motility to acid secretion. Metformin competes with histamine and serotonin for OCT1 cellular efflux, resulting in intracellular accumulation and extended hormonal messaging.^192^ Metformin also reduces the activity of diamine oxidase, an enzyme responsible for deactivating histamine to inactive metabolites, further contributing to its side effects.^192^ Ahmadi et al. utilized an animal model to show an improvement in leaky gut and subsequent intestinal inflammatory markers in response to metformin.^193^ Metformin improved lysophospholipid levels that possess demyelinating and proinflammatory properties.^194–196^ Ahmadi et al. also demonstrated an increase in metabolites that may be of benefit in the treatment of ME/CFS and LC, noting increases in bile acids, butyrate, leucine, and taurine while finding a decrease in glutamate and pyruvate.^193^

Faecal microbiome transplantation involves refining the microbiome of another person and directly inserting the sample into the target intestines. Kenyon et al. used this method to treat ME/CFS and Irritable Bowel Syndrome (IBS) by inserting donor samples transrectally into the sigmoid colon.^197^ The methodology in measuring response to therapy and transplantation technique was flawed, with current consensus favoring intrajejunal administration or delivery via colonoscopy to avoid multiple applications and increase the rate of successful retention.^198^ Using probiotics alone to improve comorbid anxiety in those with ME/CFS demonstrated success by Rao et al.’s RCT testing *Lactobacillus* administration v placebo.^199^ Stool samples collected following therapy demonstrated an unexpectedly significant rise in *Bifidobacteria*, which correlated to improved symptoms of anxiety and has been shown to be both low in ME/CFS^199,200^ and sensitive to metformin.^191^ While altering the microbiome provides a tempting target for managing symptoms of ME/CFS and LC, utilizing metformin for its microbiome impact alone may provide benefits conferred on other common comorbidities.

## Metformin and MicroRNA Modulation: Insights into Epigenetic Mechanisms

Metformin is increasingly recognized for its potential epigenetic effects, particularly its modulation of microRNAs (miRNAs).^201^ MiRNAs are small noncoding RNA molecules crucial for post-transcriptional gene regulation in various physiological processes. Studies have revealed that metformin influences the expression of several miRNAs involved in insulin signaling, apoptosis, and epithelial–mesenchymal transition (EMT). It has been shown to upregulate miRNAs like miR-26a, miR-34a, miR-146a and miR-200c,^202^ while downregulating pro-inflammatory miRNAs such as miR-155, miR-21 and miR-29a,^203,204^ thereby contributing to its anti-inflammatory properties and improved insulin sensitivity.^205^ Interestingly, miR-29a-3p has been found to be elevated in the plasma of ME/CFS patients, potentially contributing to the pathophysiology of the disease.^206,207^

In cancer research, metformin alters miRNA profiles associated with tumor suppression or oncogenic pathways, impacting cell proliferation (e.g., miR-21, miR-221) and apoptosis (e.g., let-7, miR-200 family). This suggests potential therapeutic implications in cancer treatment and prevention. Metformin’s effects on metabolic pathways, including AMPK activation and mTOR inhibition, intersect with miRNA regulation, further enhancing its metabolic benefits.

The influence of metformin on circulating miRNAs, detectable in blood and other body fluids, highlights its systemic effects. Changes in circulating miRNA profiles in response to metformin therapy could potentially serve as noninvasive biomarkers for disease management and treatment response monitoring. Understanding these epigenetic effects may lead to personalized treatment strategies in diabetes and metabolic disorders, harnessing miRNA profiles to tailor therapy. Continued research into metformin’s epigenetic effects on miRNAs holds promise for therapeutic innovations across various diseases, from cancer to cardiovascular and neurodegenerative disorders.

## Novel Treatment Strategies as a Focus for Further Research

This review has demonstrated that metformin has direct antiapoptotic and antinecrotic properties in addition to reducing cellular stress. Targeting MAC and PTP using a multiagent approach may provide for reduced side effects while increasing the robustness of the mitochondria. Potential options that target MAC and PTP include Metformin, tricyclic acid antidepressants and Propranolol at maximally tolerated doses.^187,208^ Further targets include focusing on mTORC through the direct inhibiting agent Temsirolimus augmented with an indirectly acting agent such as Metformin mediated by miRNA. This would allow a lower dose of potent chemotherapeutic agents but require phase II and phase III studies. Further evidence has been provided suggesting the role of metformin is not specifically as monotherapy in a whole of person approach, but rather one that targets the microbiome and cellular stress aspects of ME/CFS and LC.

Oral Metformin dosing for MCAS or the targeting of the sphingomyelin pathway in ME/CFS should be achievable at doses lower than 500 mg in vitro and extrapolated in vivo to measure response. Intestinal microbiota have a demonstrated role in ME/CFS and LC and represent a likely target for metformin’s methods of action. Distal small intestines should be targeted by delayed release preparations of metformin and tested against sustained release at both low and high doses to assess metformin’s microbial role in the treatment of these chronic debilitating conditions.

A multiarm randomized controlled trial of differing dose lasting a minimum of 3–4 months for each treatment cycle would be the optimal choice for testing these combinations. Due to the difference in region-specific and household-specific microbiomes, healthy controls should be sought from cohabitors. Ever changing vaccination and COVID-19 variant emergence will require optimal timing, with the trial terminating before prewinter spikes. Populations should be large enough to allow for significance in faecal metagenomic studies and comprise of dosing arms of between 500 mg daily and 2 g. Recommended analysis in addition to microbiomes should involve ATP assays or Seahorse oxygen consumption studies together with genomic, metabolomic and proteomic studies. Clinical correlation should include functional, time point-specific questionnaires coupled with bedside examination techniques. These experiments would likely yield an answer to the questions of benefit for those with ME/CFS and LC when treated with metformin and how exactly the medication is exerting its effect.

## Conclusion

Metformin acting at a biochemical level has multiple targets with which to leverage against symptoms of ME/CFS. mTOR, hypothesized to be chronically overactivated by the buildup of substrates secondary to inefficient energy manufacturing processes in those with ME/CFS and LC can be regulated by metformin. In addition to biochemical signaling pathways, direct inhibition of the electron transport chain by metformin will reduce free radicals with corresponding reduction in cellular, neurological and endovascular damage. These biochemical interactions regulated by metformin are expected to offer meaningful symptomatic relief for those suffering from ME/CFS and LC.

Studies on faecal microbiomes have revealed striking similarities in abnormalities despite the use of complex techniques. These findings offer a new potential pathway for disease treatment. Metformin, known for its impact on metabolism, can influence intestinal bacterial diversity, potentially alleviating the severity of ME/CFS and LC symptoms. Additionally, metformin may modulate immune pathways and ER stress, which could reduce mast cell degranulation in patients with comorbid MCAS and help regulate cytokine levels.

Metformin is an intriguing multiaction drug that may play a significant role in treating ME/CFS and LC. Although more research is needed to refine dosing and delivery methods, the accumulating evidence suggests it could soon be integrated into clinical practice.

## Data Availability

The data that support the findings of this study are available from the corresponding author upon reasonable request. Some data may not be made available because of privacy or ethical restrictions.

## Abbreviations

ME/CFS: Myalgic Encephalomyelitis/Chronic Fatigue Syndrome
RCT: Randomized Controlled Trial
T2DM: people with type 2 diabetes
GLUT: Glucose Transporter
AMP: Adenosine Mono Phosphate
AMPK-AMP: activated Protein Kinase
ATP: Adenosine Triphosphate
TCA: tricarboxylic acid cycle/Krebs cycle
GDP: glycerophosphate dehydrogenase
RNA: ribose nucleic acid
mGPDH: Mitochondrial glycerol-3-phosphate dehydrogenase
EBV: Epstein–Barr Virus
PBMC: Peripheral Blood Mononuclear Cells
LC: Long Covid
ACE: Angiotensin Converting Enzyme
mTOR: Mamilian/Mechanistic Target of Rapamycin
IL: Interleukin
CX: Chemokine
NLR: neutrophil to lymphocyte ratio (NLR)
NLRP3: NOD-like receptor family, pyrin domain containing 3
HHV8: Kaposi Sarcoma associated herpesvirus
HMGCR: HMGCoA reductase
PI3K: Phosphoinositide 3-kinases
MCAS: Mast Cell Activation Syndrome
AHR: Aryl hydrocarbon receptor
ARDS: Acute Respiratory Distress Syndrome
SCFA: short chain fatty acids
FXR: farsenoid X receptor blockade
ER: endoplasmic reticulum
GSK: glycogen synthase kinase
ASD: autism spectrum disorders
FIQ: fatigue inventory questionnaire
NMR: Nuclear Magnetic Resonance
